# Effect of nitrogen fertilizer regulation on rice panicle morphology, yield and economic benefits under low-temperature stress

**DOI:** 10.3389/fpls.2025.1720280

**Published:** 2025-12-19

**Authors:** Xiaojing Du, Yanhong Zhang, Tianyu Hou, Zhiqiang Zhao, Dong Li, Mintai Kang, Xiaorong Wen, Fusen Tang, Buhaliqem Abliz, Yuhong Qi, Quan Yuan, Jie Yuan, Fengbin Wang

**Affiliations:** Research Institute of Xinjiang Uygur Autonomous Region Academy of Agricultural Sciences/Northwest Center of National Saline-alkali Tolerant Rice Technology Innovation Center, Urumqi, China

**Keywords:** rice, low-temperature stress, nitrogen, panicle traits, grain yield, economic benefits

## Abstract

**Introduction:**

This study aimed to determine the optimal nitrogen (N) application rate for mitigating low-temperature (LT) stress damage at different growth stages and to elucidate the underlying mechanisms in rice cultivation in Xinjiang.

**Methods:**

A split-plot field experiment was conducted in 2024 using the rice cultivar ‘Xinjing 8’. The main plots included three temperature environments: control (CK), lowtemperature stress at the tillering (TLS), and low-temperature stress at the jointing stage (JLS), perform a 3-day LT treatment at 16°C. Subplots were assigned five N application rates: 295.5, 267.9, 240.3, 212.7, and 0 kg·ka^-1^. The effects of N rate on rice panicle traits, yield, and economic benefits under these growing environments were evaluated.

**Results:**

The results indicated that under lowtemperature stress, rice exhibited significant reductions in panicle weight, plant height, number of secondary branches, effective panicles, and grain yield per panicle, particularly during the jointing stage. Nitrogen supplementation under low-temperature stress significantly improved rice tillering and panicle traits, simultaneously elevating grain yield and economic benefits. Specifically, at optimal nitrogen application rate of 267.9 kg ha^-1^, the crop yields under TLS and JLS treatments were 4424.40 and 2350.82 kg ha^-1^. At the same N application rate, a positive net income was achieved only in the CK treatment.

**Discussion:**

This study identifies a nitrogen application rate of 267.9 kg ha^-1^ as an effective measure to mitigate low-temperature stress-induced limitations on rice panicle development, resulting in enhanced yield and economic gains. Consequently, it provides a practical solution for advancing sustainable rice cultivation in coldprone regions such as Xinjiang.

## 1 Introduction

Rice (*Oryza sativa* L.) is one of the most important global food crops, serving as a staple food for nearly half of the world’s population. However, with the continuous growth of the global population, it is estimated that by 2050, rice production must double compared to 2005 to meet the increasing food demand (Verma et al., 2021). Recently, global warming has markedly shifted the temperature variability and extremes around the world, and the occurrence of periodic low-temperatures (LT) may increase (Shen et al., 2018). China has the world’s second largest rice cultivation area and the world’s highest rice production, accounting for 29.9% of global production. Abnormal chilling events increase risks and losses to rice production, decrease agricultural income stability, and thereby threaten food supply stability. Therefore, exploring strategies to mitigate the impact of low-temperature on yield loss and improving the efficiency of low-temperature prevention and control for rice in China is of critical research significance for ensuring China’s food security (Shimono et al., 2007).

Rice originated in tropical regions (Yuan et al., 2021), and exhibits a preference for warm environments, with an optimal growth temperature range is 25-32°C, while being highly sensitive to low-temperature stress (Ma et al., 2024). Low-temperature affect rice starting from seed germination until physiological maturity. If rice seedlings encounter low-temperatures after spring sowing, they may exhibit slow growth, yellowing, poor development, wilting, and even death, seriously affecting rice growth, development, and final yield (Jia et al., 2022b). During the vegetative growth period, Low-temperatures dropping below 15°C can inhibit the differentiation of tiller primordia, reducing the number of effective tillers by 40-60% (Guo et al., 2022). Low-temperature usually exacerbates the balance between the source of energy and the metabolic sink, which inhibits the rate of photosynthesis and results in reduced green leaf area and biomass accumulation (Shi et al., 2022a). Low-temperatures during growth stages of booting result in pollen sterility, delayed and inadequate heading, and restricted anther dehiscence (Ali et al., 2021; Gao, et al., 2024; Shi et al., 2022b), which can lead to a 30% to 40% reduction in rice yield, and in extreme cases, even total crop failure. Low-temperatures at the rice grain filling stages delays the rice maturity date and cause frost disasters, which ultimately leads to insufficient grain filling, rice grain weight decrease and yield reduction, along with significant alterations in amylose and protein content in rice grains (Guo et al., 2024). Researchers have been working on screening rice varieties with enhanced cold tolerance (Lee et al., 2025; Nie et al., 2020). However, large-scale adoption of such varieties remains challenging. Most existing studies on low-temperature stress in rice have focus on the Northeast China (Ma et al., 2023; Shi et al., 2023). Xinjiang, characterized by a typical arid and semi-arid continental climate, achieves a rice yield per unit area that is 35.18% higher than the national average (National Statistical Office, 2024). However, research on the effects of low-temperatures on rice in this region remains relatively limited. In production practice, nitrogen fertilizer is usually applied when rice leaves show yellow due to low-temperatures, aiming to promote tillering and improve yield (Wang et al., 2016). Yet, excessive nitrogen application after temperature returns to normal can lead to redundant tillering, prolong the vegetative growth phase, and delay maturity (Peng et al., 2018). It is uncertain whether nitrogen application under low-temperature conditions is effective in supporting the recovery and normal growth of rice.

This study focused on the critical growth stages of tillering and jointing for rice. Based on the experimental data for Nitrogen application treatments, we compared the response of yield traits to chilling treatment at different growth stages for the selected rice variety. This study was conducted with the following objectives: (1) To evaluate the effects of low-temperature (LT) stress at critical vegetative stages on rice panicle morphology, yield components, and economic returns. (2) To identify the optimal nitrogen (N) application rate for alleviating LT stress and maximizing productivity and profitability in Xinjiang rice production systems. (3) To provide a scientific basis for developing defensive strategies against LT stress and rational N management practices in cold-affected seasons.

## 2 Materials and methods

### 2.1 Overview of the experimental site

**Figure 1:**
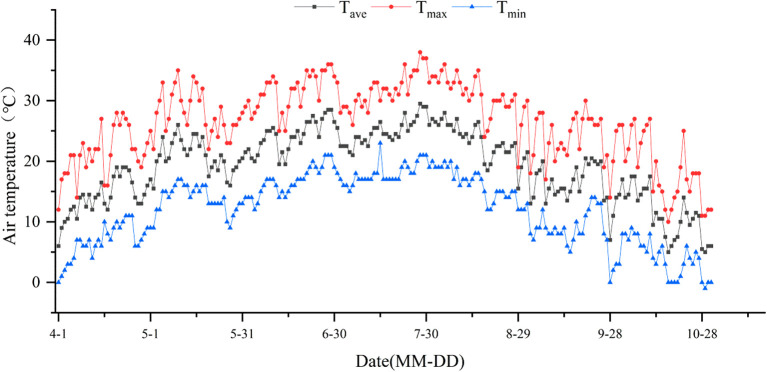
Shows the air temperature during the rice growing period (April to October) in 2024. “MM” is the month and “DD” is the day. The same as below.

This experiment was conducted from April to October 2024 at the Chabuchar Rice Experiment Station of Xinjiang Academy of Agricultural Sciences (43°50′ N, 81°7′ E). The region is characterized by an average annual sunshine duration of 2810.4-2846.0 hours, an average annual precipitation of 222 mm, and a frost-free period ranging from 169 to 177 days. It experiences cold winters and hot summers, with a mean annual temperature of 7.9 °C. The soil at the experimental site is clayey, with the following physicochemical properties (0–30 cm depth): pH 8.0, electrical conductivity 144 mS·cm^-^¹, organic matter 20.11 g·kg^-^¹, total nitrogen 1.65 g·kg^-^¹, available nitrogen 113.40 mg·kg^-^¹, available phosphorus 36.25 mg·kg^-^¹, and available potassium 124.20 mg·kg^-^¹. Temperature variations during the experimental period (April–October 2024) are presented in **Figure 1**.

### 2.2 Test materials

The experimental material employed in this study was the rice cultivar ‘Xinjing 8’, which was developed locally by the Xinjiang Academy of Agricultural Sciences. Nitrogen, phosphorus, and potassium were supplied in the form of urea (N ≥ 46%; Xinjiang, China), double superphosphate (46% P_2_O_5_; Yunnan, China), and potassium sulfate (K_2_O ≥ 50%; Qinghai, China), respectively.

### 2.3 Experimental design

A split-plot design with two factors was employed. The main-plot factor consisted of three temperature environments: low-temperature treatment at the tillering stage (TLS), low-temperature treatment at the jointing stage (JLS), and conventional field conditions as the control (CK). The sub-plot factor involved five nitrogen (N) application rates based on side-deep fertilization technology. Previous studies indicated that an annual application of 295.5 kg·ha^-^¹ (N) could achieve high yields under local conditions. Based on this rate, the following five N levels were set: 100% (N_295.5_), 80% (N_267.9_), 60% (N_240.3_), 40% (N_212.7_), and a zero-N control (N_0_), resulting in a total of 15 treatment combinations.

Cold tolerance test method: At the time of transplanting, 9 plants of each treatment to be tested were transplanted into individual pots (10 cm×10 cm) respectively, and labeled. After transplanting, they were placed in the field under the same water and fertilizer management as the field control. At the tillering or jointing stages, they were transferred to an artificial climate chamber for low-temperature treatment for 3 days, with the constant temperature set at 16°C and the light duration at 16 hours. After 3 days, the pots were removed, and the plants were buried in the field until maturity. The field control plants were labeled but received no low-temperature treatment. Phosphorus (P) and potassium (K) fertilizers were applied uniformly across all treatments. Phosphorus was applied as a one-time basal dose at 138 kg·ha^-^¹ (P_2_O_5_). Potassium was split-applied, with 75 kg·ha^-^¹ (K_2_O) as basal fertilizer and 37.5 kg·ha^-^¹ (K_2_O) as panicle fertilizer. Detailed nitrogen application schedules for each growth stage are provided in **Table 1**.

**Table 1:** Nitrogen application rates for different treatments(kg·ha^-1^).

Treatment	Base fertilizer	Regreening stage	Early tillering stage	Tillering peak period	Full heading date	Total
N_295.5_	157.50	0.00	55.20	41.40	41.40	295.50
N_267.9_	157.50	0.00	44.16	33.12	33.12	267.90
N_240.3_	157.50	0.00	33.12	24.84	24.84	240.30
N_212.7_	157.50	0.00	22.08	16.56	16.56	212.70
N_0_	0.00	0.00	0.00	0.00	0.00	0.00

### 2.4 Field management

Bowl-shaped blanket seedling trays were used for seedling raising. During transplantation, side-deep fertilization was simultaneously applied using a Yanmar YR60D (2ZGQ-60D; Jiangsu, China) rice transplanter. With this technology, both the base and tillering fertilizers were placed 3 cm to the side and 5 cm deep relative to the rice roots. For transplanting cultivation, the seedlings were raised in the nursery for 28 days before being transplanted into the field with a row spacing of 30 cm and a distance of 14 cm between hills. A large-plot experimental design was employed to accommodate mechanized transplanting operations. Each treatment was assigned to a 90 m^2^ main plot, which was subdivided into three 30 m^2^ (10 m × 3 m) sub-plots. Ridges measuring 30 cm in height and 50 cm in width were constructed between sub-plots and covered with plastic film to enable separate irrigation and drainage, thereby preventing water and fertilizer interaction among adjacent experimental units. All other field management practices followed local commercial production standards.

### 2.5 Measured items and methods

#### 2.5.1 Growth stages recording

The dates of each growth stage of rice (sowing period, transplanting period, heading period, full heading period, and maturity period) were accurately recorded based on the standard for each growth period of rice (Agricultural Industry Standard of the People's Republic of China, 2021).

#### 2.5.2 SPAD value

Starting from 30 days after transplanting, the SPAD (Soil Plant Analysis Development) values of rice leaves were measured at 10-day intervals. On each sampling date, between 11:00 and 13:30 under clear and windless conditions, nine uniformly growing hills per plot were selected. The chlorophyll content of the uppermost fully expanded leaf (flag leaf) was measured using a hand-held SPAD-502 Plus chlorophyll meter (produced by Konica Minolta, Japan), and the average value per plot was calculated.

#### 2.5.3 Tiller dynamics

Seven days after transplanting, nine representative hills in each plot were randomly selected and tagged. The number of tillers per hill was counted every 7 days until the full heading stage. Data on basic seedlings, maximum seedling number, and effective panicle number were recorded. The tillering rate and panicle-forming rate (effective tiller percentage) were calculated as follows:


Tillering rate (%)=(Maximum number of plants per unit area−Basic number of plants per unit area)/Basic number of plants per unit area×100%

Panicle setting rate(%)=(Number of effective panicles/Maximum number of plants)×100%

#### 2.5.4 Yield component analysis and grain quality measurement

Before the rice harvest, the number of effective panicles in 9 rice clusters was investigated in each plot. After harvesting and threshing, the samples were air-dried to a constant weight for yield component analysis. The following parameters were determined: panicle length, weight per panicle, number of primary branches, number of secondary branches, number of effective panicles, number of grains per panicle, number of filled grains, number of empty and shriveled grains, and 1000-grain weight, etc. Theoretical yield was calculated based on these components. Grain characteristics were measured using SC-G (Wanshen, Hangzhou, China) seed detector.

#### 2.5.5 Economic analysis

The total economic input (expressed in CNY·ha^-^¹) was calculated by aggregating the costs associated with all cultivation factors. These included expenditures on seeds, nursery trays, substrate, agricultural film, irrigation, personnel labor, fertilizers and agrochemicals, mechanical operations, and other incidentals. All cost data were acquired through local market surveys. The average market price of rice was taken as 3.00 CNY·kg^-^¹, and the currency conversion rate used was 1.00 USD = 7.11 CNY. The output income, net income, input-output ratio were calculated as followed:


$$\text{Output income }(\text{CNY}\cdot {\text{ha}}^{-1})=\text{Grain yield}\times \text{Grain price}$$

$$\text{Net income}\;(\text{CNY}\cdot {\text{ha}}^{-1})=\text{Output income}-\text{Total imput}$$

Input−output ratio=Output income/Total imput

### 2.6 Data processing

Data processing was conducted using Microsoft Excel 2010, graphs were made with Origin 2022. The data were subjected to variance analysis using SPSS 26.0 to determine the treatment effects on the agronomic traits. The differences between means were determined using the least significant difference test at *P* < 0.05. For comparisons under a specific temperature treatment, Duncan’s multiple range test was applied to identify significant differences in nitrogen uptake.

## 3 Results

### 3.1 Impact of low-temperature on agronomic traits of rice under different nitrogen application rates

#### 3.1.1 Phenological development

Under consistent environmental conditions, the first heading date, full heading date and maturing date of rice are delayed with the increase of nitrogen application, and the total growth period is extended (**Table 2**). The total growth period of rice under N_295.5_ treatment was the longest, lasting 173 days (CK), 175 days (TLS) and 178 days (JLS) respectively, and it was prolonged by 1–13 days compared to other nitrogen application treatments. Under the same nitrogen application treatment, the total growth period of JLS was extended by 1–3 days compared to TLS. Specifically, the N_267.9_ treatment showed only a 1-day extension, whereas all other treatments exhibited a 3-day extension.

**Table 2:** Effects of low-temperature on phenological period of rice with different nitrogen application rates.

Treatment		Seeding date/(MM-DD)	Transplanting date/(MM-DD)	First heading date/(MM-DD)	Full heading date/(MM-DD)	Maturing date/(MM-DD)	Growth duration/d
N_295.5_	CK	4-10	5-8	7-23	7-27	9-30	173
	TLS	4-10	5-8	7-25	7-28	10-2	175
	JLS	4-10	5-8	7-28	8-1	10-5	178
N_267.9_	CK	4-10	5-8	7-22	7-25	9-29	172
	TLS	4-10	5-8	7-22	7-26	10-1	174
	JLS	4-10	5-8	7-23	7-27	10-2	175
N_240.3_	CK	4-10	5-8	7-21	7-24	9-26	169
	TLS	4-10	5-8	7-23	7-26	9-28	171
	JLS	4-10	5-8	7-29	7-30	10-1	174
N_212.7_	CK	4-10	5-8	7-20	7-23	9-24	167
	TLS	4-10	5-8	7-21	7-25	9-26	169
	JLS	4-10	5-8	7-28	7-31	9-29	172
N_0_	CK	4-10	5-8	7-16	7-20	9-17	160
	TLS	4-10	5-8	7-19	7-22	9-19	162
	JLS	4-10	5-8	7-23	7-26	9-22	165

“MM” is the month and “DD” is the day. CK: conventional field conditions as the control, TLS: low-temperature treatment at the tillering stage, JLS: low-temperature treatment at the jointing stage. The same as below.

#### 3.1.2 SPAD

**Figure 2:**
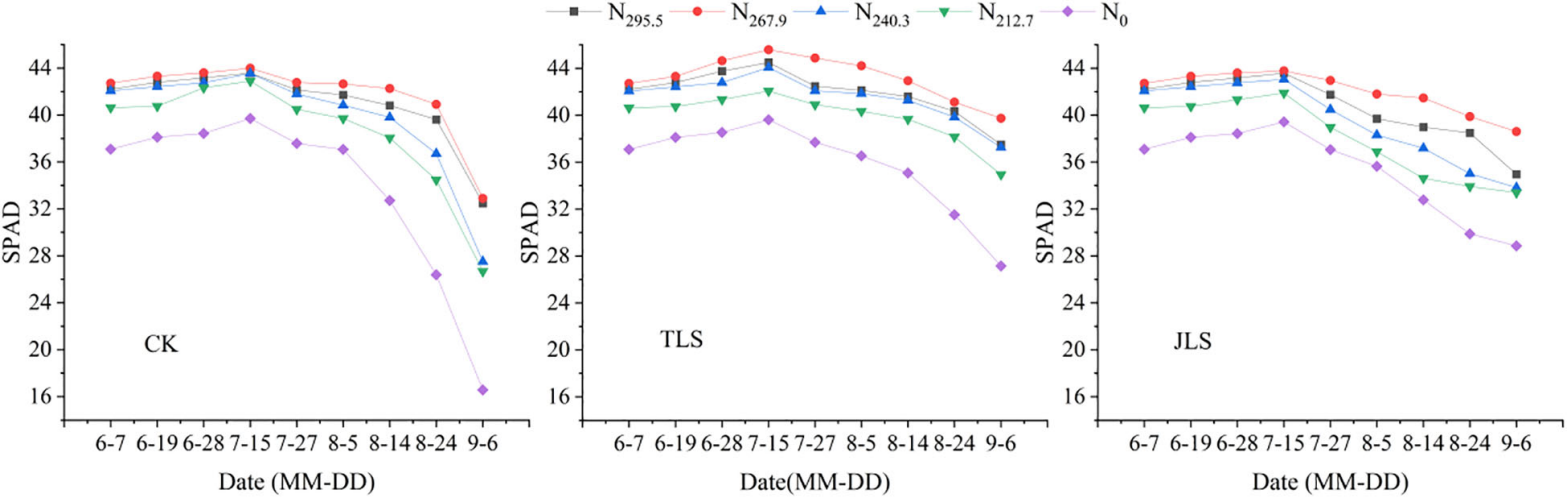
Effect of low-temperature on chlorophyll SPAD value in rice with different nitrogen application rates. CK, conventional field conditions as the control; TLS, low-temperature treatment at the tillering stage; JLS, low-temperature treatment at the jointing stage.

Under the same environmental conditions, the SPAD values of rice leaves under different nitrogen application rates exhibited an initial increase followed by a decreasing trend throughout the growth cycle (**Figure 2**). The SPAD values of rice leaf blades reached the highest on July 15^th^, with the maximum under the N_267.9_ treatment being 43.99 (CK), 45.58 (TLS), and 43.77 (JLS), respectively. The overall trend indications were N_267.9_ > N_295.5_ > N_240.3_ > N_212.7_ > N_0_. Under the same nitrogen application treatment, the decline in leaf SPAD values TLS and JLS was less than that of CK, with the smallest decline occurring JLS.

#### 3.1.3 Tillering rate and spike rate

**Figure 3:**
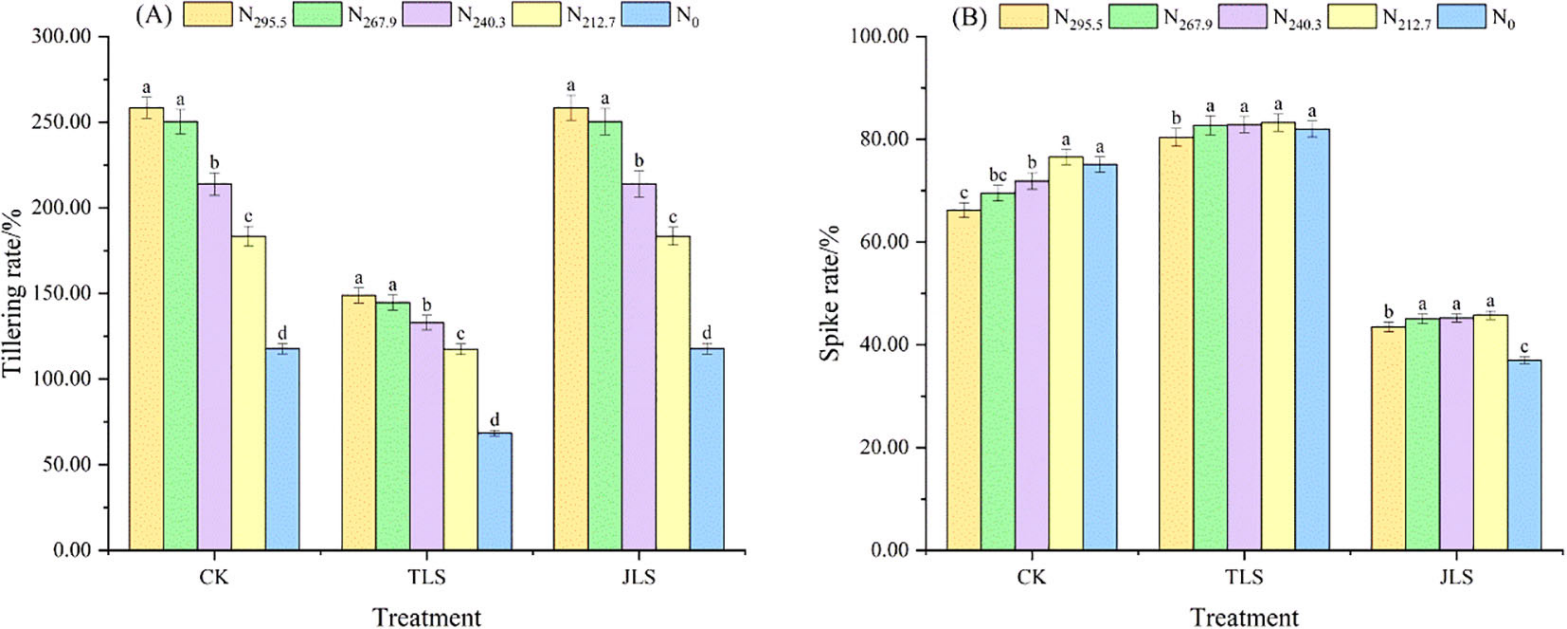
Effects of low-temperature on tillering rate and spike rate of rice with different nitrogen application rates. Different letters in lowercase indicate significant difference of the data in the same temperature stress treatment at *P* < 0.05. Data are the means ± standard deviation (means ± SD). **(A)** Tillering rate; **(B)** Spike rate.

Under consistent nitrogen application rates, the tillering rate of rice followed a similar trend in both CK and JLS, with both being significantly higher than that in TLS. Notably, the tillering rate in TLS was on average 40.19% lower than that in JLS and the CK (**Figure 3A**). In contrast, the spike rate of rice in TLS increased by an average of 89.89% and 14.14%, respectively, compared to JLS and CK (**Figure 3B**).

Under the same environmental conditions, the tillering rate of rice decreased with reduced nitrogen application. The highest tillering rate was observed under the N_295.5_ treatment. However, the difference between N_267.9_ and N_295.5_ treatment was not significant, though both differed significantly from other nitrogen treatments (*P*<0.05). Meanwhile, the spike rate exhibited an initial increase followed by a decrease as nitrogen application was reduced, with the maximum spike rate recorded under the N_212.7_ treatment.

#### 3.1.4 Plant height and stem diameter

**Figure 4:**
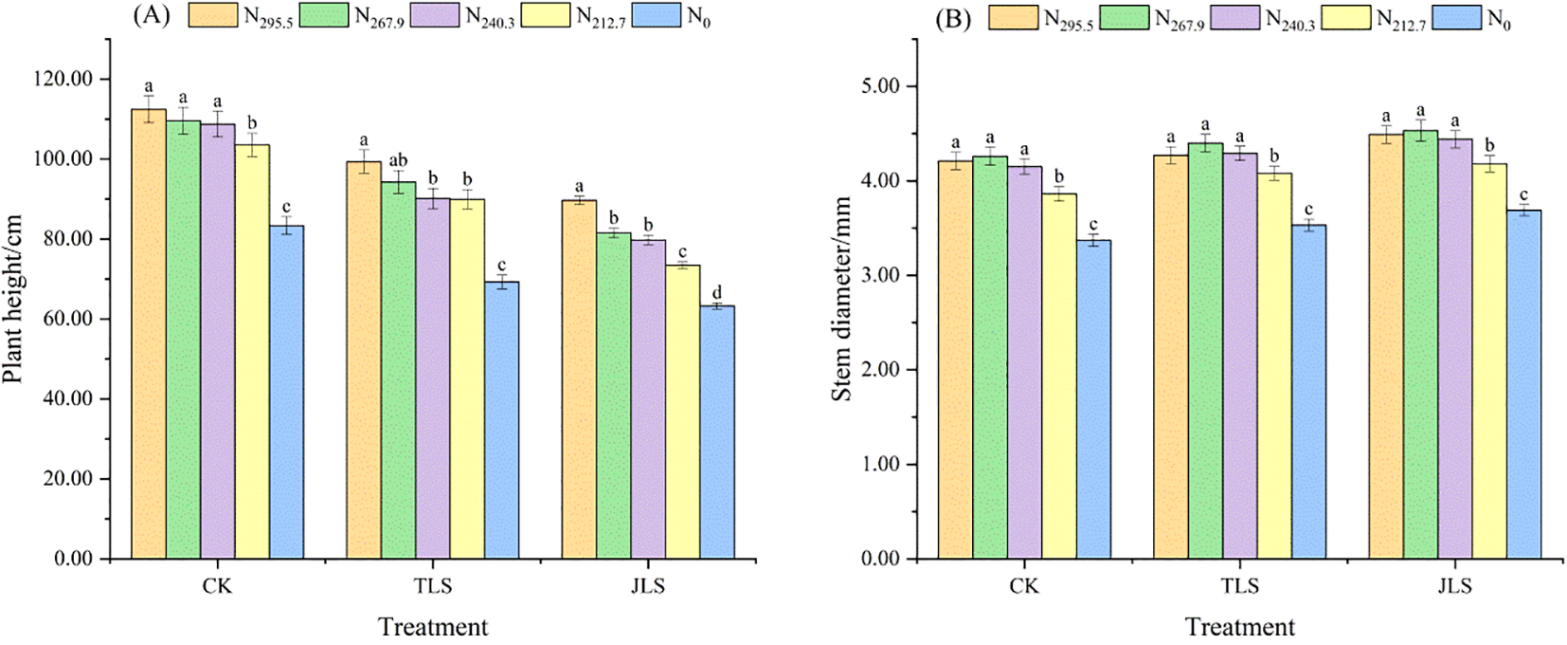
Effects of low-temperature on plant height and stem diameter of rice with different nitrogen application rates. Different letters in lowercase indicate significant difference of the data in the same temperature stress treatment at P <0.05. Data are the means ± standard deviation (means ± SD). **(A)** Plant height; **(B)** Stem diameter.

Under consistent nitrogen application rates, rice plant height followed the order: CK > JLS > TLS (**Figure 4A**). In contrast, the stem diameter of rice was exactly the opposite, with JLS being the thickest and CK the thinnest (**Figure 4B**). Under the same environmental conditions, as the nitrogen application rate decreases, the plant height of rice plants decreases accordingly, while the stem thickness of rice shows a trend of increasing first and then decreasing. Among them, the stem diameter reached the peak under the treatment of N_267.9_, which was 4.26 mm (CK), 4.40 mm (TLS), and 4.53 mm (JLS), respectively. Notably, there was no significant difference between the N_267.9_ treatment and the N_295.5_ and N_240.3_ treatments, while significant differences were detected compared to the N_212.7_ and N_0_ treatments.

### 3.2 Effects of low-temperature on panicle morphology of rice with different nitrogen application rates

#### 3.2.1 Panicle length and single spike weight

**Figure 5:**
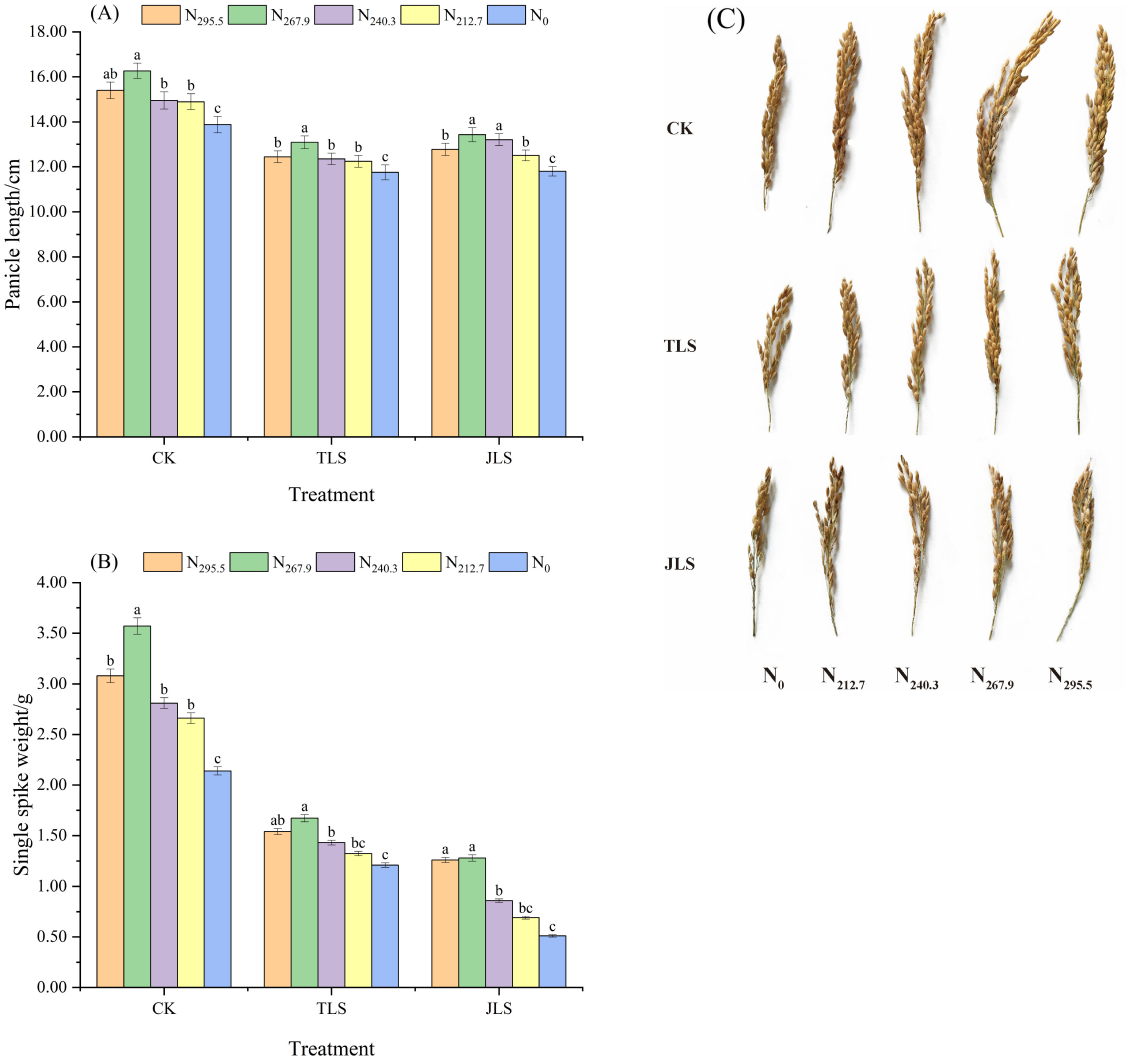
Effects of low-temperature on panicle length and single spike weight of rice with different nitrogen application rates. Different letters in lowercase indicate significant difference of the data in the same temperature stress treatment at *P* <0.05. Data are the means ± standard deviation (means ± SD). **(A)** Panicle length; **(B)** Single spike weight. **(C)** Rice panicle morphology.

When the nitrogen application rate was consistent, relative to CK, the panicle lengths of TLS and JLS exhibited average reductions of 17.93% and 15.49%, respectively (**Figure 5A**). A similar trend was observed for the single spike weight, which average decreased by 67.74% and 51.54%, respectively. These findings indicate that low-temperature adversely affects the morphological development of rice panicles, especially with a significant reduction in the weight per panicle (**Figure 5B**).

Under the identical environmental conditions, both panicle length and single panicle weight of rice exhibited a trend of “increasing first and then decreasing” with the reduction of nitrogen application rate and were the largest under the N_267.9_ treatment. These findings suggest that under low-temperature conditions, an appropriate nitrogen application level is conducive to increasing panicle length and single panicle weight, whereas excessive nitrogen may inhibit their growth and development (**Figure 5C**).

#### 3.2.2 Number of branches

**Figure 6:**
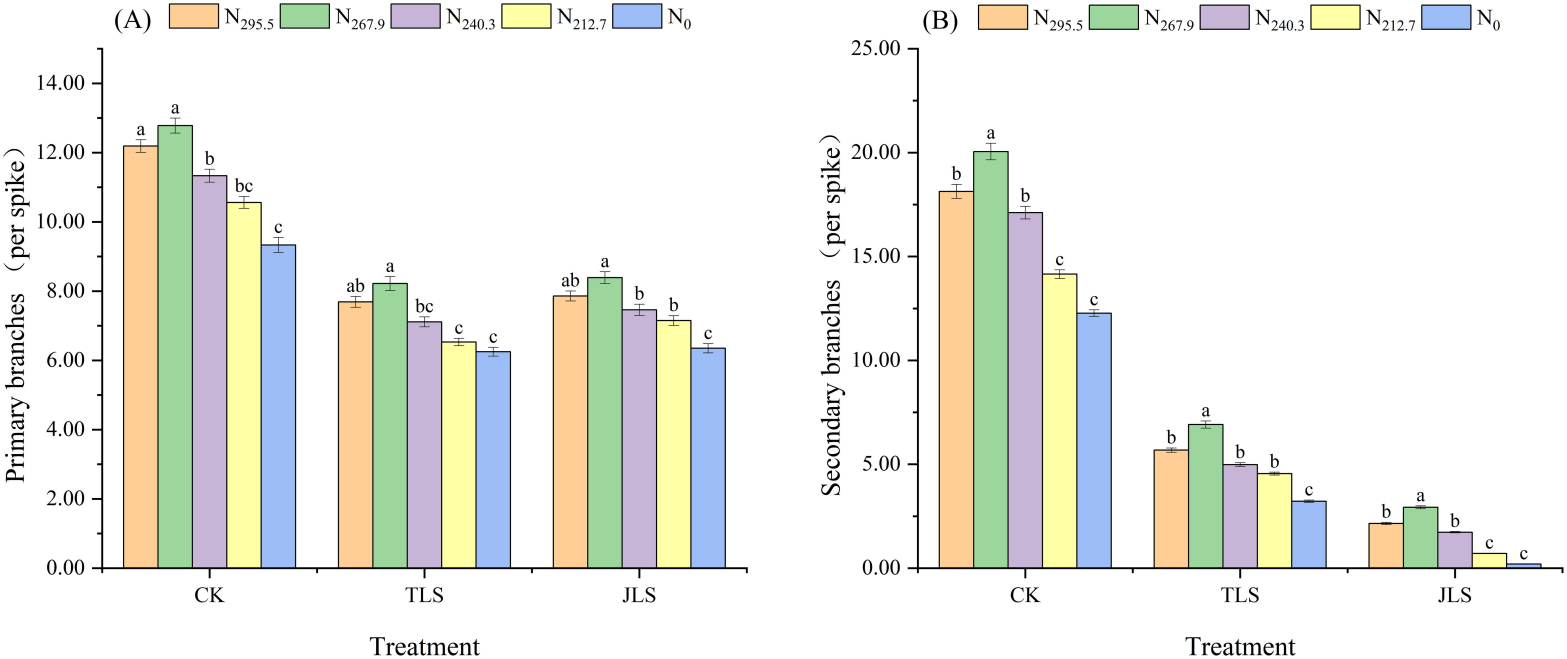
Effects of low-temperature on number of branches with different nitrogen application rates. Different letters in lowercase indicate significant difference of the data in the same temperature stress treatment at *P* <0.05. Data are the means ± standard deviation (means ± SD). **(A)** Primary branches; **(B)** secondary branches.

When the nitrogen application rate was consistent, the number of primary branches and secondary branches in CK was significantly higher than that of TLS and JLS (**Figure 6**). Among them, there was no significant difference in the primary branches between TLS and JLS. The number of secondary branches in TLS was significantly higher than that in JLS (*P* < 0.05). Under the same environmental conditions, the number of primary branches and secondary branches of rice exhibited a trend of “increasing first and then decreasing” with a reduction in nitrogen application rate, with the largest values recorded in the N_267.9_ treatment. Among them, the number of secondary branches was 22.05 per panicle (CK), 7.91 per panicle (TLS), and 2.93 per panicle (JLS), respectively. This indicates that CK had 178.76% and 652.56% more secondary branches than TLS and JLS, respectively.

#### 3.2.3 Grain morphology of rice

Under consistent nitrogen application rates, grain area, circumference, length, width, and diameter all followed the order: CK > JLS > TLS, with JLS showing a significant reduction in these parameters (**Table 3**). In contrast, the grain length-to-width ratio exhibited an opposite trend. Specifically, the length-to-width ratio of JLS increased significantly by 6.77% and 6.88% compared to CK and TLS, respectively. Additionally, grain roundness in JLS was significantly lower than that in CK and TLS.

**Table 3:** Effects of low-temperature on grain traits of rice with different nitrogen application rates.

Traits	Treatment	N_295.5_	N_267.9_	N_240.3_	N_212.7_	N_0_
Grain area /mm^2^	CK	17.185 ± 0.081 Ab	17.029 ± 0.125 Ab	17.205 ± 0.162 Ab	17.355 ± 0.196 Ab	17.759 ± 0.240 Aa
	TLS	16.903 ± 0.028 Bb	16.757 ± 0.076 Bb	16.978 ± 0.109 Ab	16.912 ± 0.138 Ab	17.727 ± 0.216 Aa
	JLS	15.334 ± 0.071 Cc	15.157 ± 0.024 Cc	15.648 ± 0.181 Bb	15.835 ± 0.168 Bb	17.057 ± 0.273 Ba
Grain circumference /mm	CK	18.833 ± 0.075 Aa	18.727 ± 0.103 Aa	18.817 ± 0.052 Aa	18.867 ± 0.200 Aa	18.891 ± 0.126 Aa
	TLS	18.701 ± 0.045 Bb	18.700 ± 0.104 Ab	18.643 ± 0.093 Bb	18.819 ± 0.065 Ab	19.136 ± 0.132 Aa
	JLS	18.493 ± 0.020 Cb	18.125 ± 0.022 Bd	18.260 ± 0.090 Ccd	18.367 ± 0.046 Bbc	18.936 ± 0.137 Aa
Ratio of length to width for grain	CK	2.716 ± 0.011 Ba	2.715 ± 0.012 Ba	2.708 ± 0.016 Ca	2.705 ± 0.029 Ba	2.633 ± 0.013 Cb
	TLS	2.675 ± 0.033 Bab	2.726 ± 0.066 Ba	2.634 ± 0.015 Bb	2.711 ± 0.019 Ba	2.715 ± 0.018 Ba
	JLS	3.007 ± 0.045 Aa	2.900 ± 0.010 Ab	2.847 ± 0.022 Ab	2.850 ± 0.041 Ab	2.784 ± 0.038 Ac
Grain length /mm	CK	7.782 ± 0.026 Aa	7.737 ± 0.042 Aa	7.773 ± 0.025 Aa	7.788 ± 0.084 Aa	7.779 ± 0.051 Ba
	TLS	7.670 ± 0.027 Bbc	7.683 ± 0.072 Abc	7.623 ± 0.028 Bc	7.747 ± 0.039 Ab	7.911 ± 0.055 Aa
	JLS	7.670 ± 0.027 Bb	7.533 ± 0.028 Bc	7.565 ± 0.029 Cc	7.595 ± 0.045 Bc	7.855 ± 0.057 ABa
Grain width /mm	CK	2.877 ± 0.011 Abc	2.86 ± 0.006 Ac	2.882 ± 0.019 Abc	2.902 ± 0.041 Ab	2.973 ± 0.024 Aa
	TLS	2.883 ± 0.021 Abc	2.85 ± 0.023 Ac	2.911 ± 0.010 Aab	2.865 ± 0.009 Ac	2.931 ± 0.022 Aa
	JLS	2.576 ± 0.026 Bd	2.624 ± 0.006 Bc	2.683 ± 0.022 Bb	2.690 ± 0.025 Bb	2.840 ± 0.031 Ba
Grain diameter /mm	CK	4.671 ± 0.010 Ab	4.649 ± 0.018 Ab	4.673 ± 0.023 Ab	4.694 ± 0.057 Ab	4.749 ± 0.034 Aa
	TLS	4.634 ± 0.004 Bbc	4.608 ± 0.015 Bc	4.643 ± 0.016 Ab	4.634 ± 0.005 Abc	4.743 ± 0.028 Aa
	JLS	4.410± 0.027 Ccd	4.381 ± 0.001 Cd	4.453 ± 0.028 Bbc	4.482 ± 0.015 Bb	4.653 ± 0.038 Ba
Grain roundness /mm	CK	0.384 ± 0.001 Bb	0.384 ± 0.002 Ab	0.385 ± 0.003 Bb	0.387 ± 0.002 Ab	0.398 ± 0.001 Aa
	TLS	0.393 ± 0.004 Aab	0.387 ± 0.005 Ac	0.399 ± 0.002 Aa	0.388 ± 0.003 Abc	0.386 ± 0.002 Bc
	JLS	0.345 ± 0.005 Cc	0.361 ± 0.003 Bb	0.367 ± 0.002 Cb	0.365 ± 0.005 Bb	0.376 ± 0.004 Ca

Different lowercase letters in the same row indicate significant differences between nitrogen application treatments (*P* < 0.05), and different uppercase letters in the same column indicate significant differences between low-temperature treatments (*P* < 0.05).

Under identical environmental conditions, all grain dimensional traits (area, circumference, length, width, and diameter) were highest under the N_0_ treatment. The grain length-to-width ratio peaked under the N_295.5_ treatment in both CK and JLS, with values of 2.716 and 3.007, respectively. In TLS, however, the maximum length-to-width ratio was observed under the N_267.9_ treatment (2.726), which showed a significant difference only when compared to the N_240.3_ treatment (*P* < 0.05).

### 3.3 Impact of low-temperature on rice yield and economic benefits under different nitrogen application rates

#### 3.3.1 Yield and yield components

Under consistent nitrogen application rates, grain yield, effective panicle number, total grain number per panicle, seed setting rate, and 1000-grain weight all followed the order: CK > JLS > TLS (**Table 4**). Among these traits, effective panicle number and grain yield showed significant differences among the three treatments. With the exception of the N_0_ treatment, no significant difference was observed in total grain number per panicle between TLS and JLS. In addition, no significant difference was detected in seed setting rate or 1000-grain weight between CK and TLS, although both were significantly higher than JLS.

**Table 4:** Effects of low-temperature on rice yield and yield components at different nitrogen application rates.

Traits	Treatment	N_295.5_	N_267.9_	N_240.3_	N_212.7_	N_0_
Effective panicle number/(10^4^·ha^-1^)	CK	392.86 ± 11.07 Aa	408.57 ± 13.80 Aa	380.95 ± 11.84 Aab	364.05 ± 11.98 Ab	270.71 ± 9.15 Ac
	TLS	330.00 ± 11.39 Ba	340.71 ± 10.59 Ba	325.86 ± 10.44 Bab	303.57± 9.20 Bb	228.57 ± 7.14 Bc
	JLS	255.57 ± 8.83 Cab	265.29 ± 9.42 Ca	239.67 ± 8.22 Cbc	217.62 ± 7.23 Cc	133.33 ± 4.25 Cd
Spikelets per panicle	CK	114.47 ± 3.25 Aab	122.55 ± 4.14 Aa	116.32 ± 3.57 Ab	104.53 ± 2.94 Abc	81.50 ± 2.18 Ac
	TLS	57.19 ± 2.15 Bab	64.91 ± 2.51 Ba	49.29 ± 1.84 Bb	48.33 ± 1.75 Bb	47.03 ± 1.64 Bb
	JLS	55.69 ± 2.09 Ba	58.14 ± 2.23 Ba	46.14 ± 1.66 Bab	39.14 ± 1.36 Bbc	29.95 ± 0.99 Cc
Seed setting rate /%	CK	95.52 ± 2.38 Aa	94.74 ± 2.64 Ab	94.97 ± 2.49 Ab	95.67 ± 2.41 Aa	96.18 ± 2.39 Aa
	TLS	95.07 ± 2.53 Aa	94.51 ± 2.50 Aa	94.91 ± 2.40 Aa	95.45 ± 2.34 Aa	90.42 ± 2.34 Aa
	JLS	88.06 ± 2.26 Ba	89.84 ± 2.57 Ba	87.20 ± 2.36 Ba	83.35 ± 2.41 Bab	79.80 ± 2.06 Bb
1000-grain weight /g	CK	24.63 ± 0.67 Ab	24.71 ± 0.67 Ab	24.82 ± 0.78 Ab	24.87 ± 0.63 Ab	25.11 ± 0.67 Aa
	TLS	23.50 ± 0.68 Ab	23.52 ± 0.81 Ab	23.70 ± 0.74 Ab	24.04 ± 0.69 Aa	24.36 ± 0.66 Aa
	JLS	18.76 ± 0.72 Bc	18.85 ± 0.74 Bc	19.81 ± 0.76 Bb	20.26 ± 0.75 Bb	21.92 ± 0.78 Ba
Grain yield /(kg·ha^-1^)	CK	9522.06 ± 282.81 Ab	10549.42 ± 325.98 Aa	9400.55 ± 300.82 Ab	8148.84 ± 218.39 Ac	4795.52 ± 118.93 Ad
	TLS	3794.79 ± 133.81 Bb	4424.4 ± 154.18 Ba	3251.57 ± 102.03 Bc	3029.90 ± 89.51 Bc	2189.88 ± 62.25 Bd
	JLS	2116.12 ± 60.41 Ca	2350.82 ± 66.01 Ca	1719.23 ± 50.56 Cb	1294.52 ± 37.44 Cc	628.65 ± 18.94 Cd

Note: Different lowercase letters in the same row indicate significant differences between nitrogen application treatments (P < 0.05), and different uppercase letters in the same column indicate significant differences between low-temperature treatments (P < 0.05).

Under identical environmental conditions, grain yield, effective panicle number, and total grain number per panicle exhibited an initial increase followed by a decrease as nitrogen application was reduced, with maximum values recorded under the N_267.9_ treatment. Specifically, grain yields were 10,549.42 kg·ha^-^¹ (CK), 4,424.40 kg·ha^-^¹ (TLS), and 2,350.82 kg·ha^-^¹ (JLS), respectively. In contrast, the 1000-grain weight increased with decreasing nitrogen application, reaching its highest value under the N_0_ treatment, which differed significantly from all other nitrogen treatments (*P* < 0.05).

#### 3.3.2 Economic benefits

Under consistent nitrogen application rates, net income was positive only in the CK treatment among all fertilized groups (excluding N_0_), with an input-output ratio exceeding 1 (**Table 5**). In contrast, both TLS and JLS resulted in negative net income and input-output ratios below 1, with JLS experiencing more severe economic losses. Under identical environmental conditions, the N_0_ treatment (no nitrogen application) also yielded negative net income, indicating that nitrogen fertilization generally enhances the economic returns of rice production.

**Table 5:** Effects of low-temperature on the economic benefits of rice with different nitrogen application rates.

Traits	Treatment	N_295.5_	N_267.9_	N_240.3_	N_212.7_	N_0_
Agricultural expenses /(CNY·ha^-1^)	CK	5300.10	5180.10	5060.10	4940.10	4015.50
	TLS	5300.10	5180.10	5060.10	4940.10	4015.50
	JLS	5300.10	5180.10	5060.10	4940.10	4015.50
Mechanical fee /(CNY·ha^-1^)	CK	5250.00	5250.00	5250.00	5250.00	5250.00
	TLS	5250.00	5250.00	5250.00	5250.00	5250.00
	JLS	5250.00	5250.00	5250.00	5250.00	5250.00
Others /(CNY·ha^-1^)	CK	7200.00	7200.00	7200.00	7200.00	7200.00
	TLS	7200.00	7200.00	7200.00	7200.00	7200.00
	JLS	7200.00	7200.00	7200.00	7200.00	7200.00
Total input /(CNY·ha^-1^)	CK	17750.10	17630.10	17510.10	17390.10	16465.50
	TLS	17750.10	17630.10	17510.10	17390.10	16465.50
	JLS	17750.10	17630.10	17510.10	17390.10	16465.50
Output income/ (CNY·ha^-1^)	CK	28566.18 ± 848.42 Ab	31648.26 ± 977.93 Aa	28201.65 ± 902.45 Ab	24446.52 ± 655.17 Ac	14386.56 ± 356.79 Ad
	TLS	11384.37 ± 371.44 Bb	13273.20 ± 462.54 Ba	9754.71 ± 306.10 Bc	9089.70 ± 268.54 Bc	6569.64 ± 186.75 Bd
	JLS	6348.36 ± 181.24 Ca	7052.46 ± 198.02 Ca	5157.69 ± 151.67 Cb	3883.56 ± 112.31 Cc	1885.95 ± 56.83 Cd
Net income /(CNY·ha^-1^)	CK	10816.08 ± 848.42 Ab	14018.16 ± 977.93 Aa	10691.55 ± 902.45 Ab	7056.42 ± 655.17 Ac	-2078.94 ± 356.79 Ad
	TLS	-6365.73 ± 371.44 Bb	-4356.90 ± 462.54 Ba	-7755.39 ± 306.10 Bc	-8300.40 ± 268.54 Bc	-9895.86 ± 186.75 Bd
	JLS	-11401.74 ± 181.24 Cb	-10577.64 ± 198.02 Ca	-12352.41 ± 151.67 Cc	-13506.54 ± 112.31 Cd	-14579.55 ± 56.83 Ce
Input-output ratio	CK	1.61 ± 0.05 Ab	1.80 ± 0.06 Aa	1.61 ± 0.05 Ab	1.41 ± 0.04 Ac	0.87 ± 0.02 Ad
	TLS	0.64 ± 0.02 Bb	0.75 ± 0.03 Ba	0.56 ± 0.02 Bc	0.52 ± 0.02 Bc	0.40 ± 0.01 Bd
	JLS	0.36 ± 0.01 Ca	0.40 ± 0.01 Ca	0.29 ± 0.01 Cb	0.22 ± 0.01 Cc	0.11 ± 0.01 Cd

The average price of paddy in 2022, 2023 and 2024 is 3.00 CNY·kg^-1^. Note: Different lowercase letters in the same row indicate significant differences between nitrogen application treatments (P < 0.05), and different uppercase letters in the same column indicate significant differences between low-temperature treatments (P < 0.05).

When environmental conditions were maintained constant, total input cost increased with rising nitrogen application rates. However, output income, net income, and input-output ratio all showed an initial increase followed by a decrease as nitrogen application was reduced, with peak values observed under the N_267.9_ treatment.

## 4 Discussion

### 4.1 The impact of low-temperature on yield formation

Chlorophyll, the most critical component for photosynthesis in plants, is responsible for absorbing and converting light energy (Fotovat et al., 2007). Leaf chlorophyll content not only serves as an indicator of plant nutritional status but also reflects the crop’s tolerance to adverse stress conditions (Neufeld et al., 2006). Zhao et al. reported that low-temperature stress disrupts chlorophyll biosynthesis and chloroplast development in rice, leading to leaf yellowing or chlorosis and a significant decrease in chlorophyll content (Zhao et al., 2020). However, the present study observed higher SPAD values in later growth stages under low-temperature stress compared to field controls. This apparent contradiction may be attributed to the suspension of rice growth and development under low-temperature stress, which requires additional time for metabolic recovery and chilling damage mitigation. The extended growth duration likely allowed for compensatory chlorophyll synthesis and chloroplast repair (Lu et al., 2021), resulting in delayed phenological development but ultimately higher chlorophyll content in later stages. This aligns with findings from other studies (Li et al., 2003; Guo et al., 2024), who also reported that low-temperatures can extend the rice growth period.

The present study found that low-temperature at the jointing stage (JLS) significantly delayed both vegetative and reproductive development, extending the total growth duration. The application of appropriate nitrogen rates (N_267.9_) enhanced recovery capacity after cold damage and reduced the extension of growth days. This suggests that nitrogen nutrition plays a crucial role in modulating plant responses to low-temperature stress, possibly through its involvement in the synthesis of protective compounds, membrane components, and stress-related proteins (Ye et al., 2022). The tillering and jointing stages represent critical phases for rice vegetative growth. Most studies have shown that low-temperatures at the tillering stage reduce both tillering rate and panicle formation rate (Shimono and Okada, 2013). However, some research suggests that although low-temperature decreases tiller numbers, it may improve individual development and increase panicle formation rate (Zhang et al., 2024). The results of this study generally support the latter view. Specifically, low-temperature at the tillering stage (TLS) significantly reduced rice tillering rate and plant height but increased panicle formation rate, whereas low-temperature at the jointing stage (JLS) significantly reduced panicle formation rate. This pattern suggests that plants under stress may employ a resource optimization strategy, sacrificing tiller quantity to maintain the quality of surviving tillers. The increased panicle formation rate under TLS could result from reduced competition among fewer tillers for limited resources.

The panicle represents a critical site for rice yield formation, and its morphological development constitutes a key research focus in ideal plant-type breeding (Song et al., 2022). Panicle morphology is influenced by multiple factors, including grain number per panicle, number and length of branches, grain distribution density, and panicle length (Li et al., 2021). Most studies indicate that low-temperature stress significantly reduces the number of primary branches, secondary branches, and total branches in rice (Jia et al., 2022a). Consistent with these findings, our study observed significant reductions in panicle length, single panicle weight, and numbers of primary and secondary branches in both TLS and JLS treatments compared to CK. The more pronounced reductions in single panicle weight and secondary branch number in JLS than TLS suggest that the jointing stage represents a particularly sensitive period for panicle development. This sensitivity may be attributed to the active differentiation of panicle components during this stage, making them vulnerable to temperature stress through disruption of hormonal balance and carbohydrate partitioning (Sarma et al., 2023).

Numerous studies have demonstrated that low-temperatures reduce rice yield by decreasing effective panicle number, grains per panicle, seed setting rate, and 1000-grain weight (Huang et al., 2013; Shi et al., 2025). In our experiment, while no significant differences were observed in seed setting rate and 1000-grain weight between TLS and CK (both being significantly higher than JLS), the effective panicle number of JLS was significantly lower than that of TLS. The differential impact on yield components across developmental stages reflects the stage-specific nature of low-temperature damage. The jointing stage appears particularly crucial for determining panicle number and grain development, possibly due to its role in panicle initiation and differentiation processes (Ren et al., 2017). Consequently, the average yields of TLS and JLS decreased by 61.60% and 81.99%, respectively, compared to CK. Among treatments with the same nitrogen application rate, only CK demonstrated positive net income, while both TLS and JLS resulted in economic losses. These results indicate that low-temperature exposure during the jointing stage leads to more severe yield reduction and economic loss compared to stress during earlier growth phases. This has significant implications for rice production in temperate regions and highlights the need for targeted management strategies during sensitive growth stages. This has significant implications for rice production in subtropical regions and highlights the need for targeted management strategies during sensitive growth stages.

### 4.2 Influence of nitrogen application rate on yield formation

In this experiment, under consistent environmental conditions, the first heading date, full heading date, and maturation date of rice were progressively delayed with increasing nitrogen application rates, resulting in a corresponding extension of the total growth period. This observation aligns with previous findings that excessive nitrogen application leads to delayed crop maturity (Zhao et al., 2022). Regarding chlorophyll content, the highest nitrogen treatment (N_295.5_) resulted in a 2.79% lower SPAD value compared to the N_267.9_ treatment. This indicates that moderate nitrogen application promotes chlorophyll synthesis in rice leaves (Zhou et al., 2023), whereas excessive nitrogen may disrupt nitrogen metabolism homeostasis, potentially inducing feedback inhibition or reactive oxygen species accumulation that impairs chloroplast structure and function (Kim and Kim, 2024). This nonlinear response highlights the existence of an optimal range for nitrogen’s regulatory effect on the photosynthetic apparatus, beyond which inhibitory effects prevail (Song et al., 2023).

Nitrogen is a crucial nutrient for rice growth and development, playing a central role in tiller formation (He et al., 2024). In this experiment, a moderate increase in nitrogen fertilizer promoted tiller initiation and improved tiller quality, thereby increasing the effective panicle number and grains per panicle (Peng et al., 2018). In contrast, excessive nitrogen application suppressed the growth of tillers at the middle and lower leaf positions, reducing the number of effective tillers (Zhou et al., 2022). This suppression may be attributed to intensified light competition within the canopy and reduced photo assimilate allocation to tillers under high nitrogen conditions. Furthermore, this study found that appropriate nitrogen application under low-temperature conditions significantly promoted rice tillering, increased effective panicle number, and simultaneously enhanced both yield and nitrogen use efficiency, consistent with previous reports (Zhou et al., 2018). These results suggest that optimal nitrogen availability may alleviate the inhibitory effects of low-temperature on tiller bud germination and growth by improving the coordination of carbon and nitrogen metabolism (Liu et al., 2019; Wang et al., 2023).

Multiple studies have demonstrated that the number of primary and secondary branches in rice increases with rising nitrogen application rates (Ding et al., 2014; Zhou et al., 2017), a trend also confirmed in this study. Specifically, panicle length, single panicle weight, and branch number increased with nitrogen application up to the N_267.9_ treatment but declined under the N_295.5_ treatment, generally following the order: N_267.9_ > N_295.5_ > N_240.3_ > N_212.7_ > N_0_. This threshold response indicates that optimal nitrogen application simultaneously promotes the growth of both vegetative and reproductive organs, effectively mitigating the adverse effects of low-temperature, and enhancing cold tolerance and yield potential (Xiong et al., 2025). Conversely, excessive nitrogen may lead to insufficient carbohydrate supply to the panicles or disrupt hormonal regulation, ultimately limiting the full expression of panicle traits.

Over the past six decades, rice yields and nitrogen application rates in China have increased almost simultaneously (Zhang and Liu, 2022). However, a universally applicable optimal nitrogen rate for achieving maximum yield remains undefined. A comprehensive analysis of 514 field experiments across seven rice-producing regions in Hubei Province demonstrated that yields in nitrogen-fertilized plots were significantly higher than in non-fertilized plots, with medium nitrogen treatment yielding the best results (Wang et al., 2012). Numerous studies have reported that excessive nitrogen application can lead to over-luxuriant growth and delayed maturation, impairing grain filling, reducing the seed-setting rate and 1000-grain weight, and ultimately causing yield loss (Chen et al., 2022; Huang et al., 2020). Under identical environmental conditions in this study, effective panicle number, total grains per panicle, yield, net income, and input-output ratio all exhibited an initial increase followed by a decrease as nitrogen application was reduced, peaking under the N_267.9_ treatment. Specifically, yields were 10,549.42 kg·ha^-1^ (CK), 4,424.40 kg·ha^-1^ (TLS), and 2,350.82 kg·ha^-1^ (JLS), respectively. Under CK conditions, output income, net income, and input-output ratio reached 31,648.26 CNY·ha^-1^, 14,018.16 CNY·ha^-1^, and 1.80, respectively. Notably, net income under the N_267.9_ treatment increased by 29.60%, 31.11%, and 98.66% compared to the N_295.5_, N_240.3_, and N_212.7_ treatments, respectively. These results clearly demonstrate that excessive nitrogen application under low-temperature conditions at both the tillering (TLS) and jointing (JLS) stages is detrimental to achieving high yield and income in rice production, while also resulting in significant nitrogen wastage (Ai et al., 2024), a conclusion consistent with observations under normal temperature conditions.

Rice yield is primarily determined by effective panicle number, with secondary contributions from grains per panicle, seed-setting rate, and 1000-grain weight (Bahmutsky et al., 2024). This study also found that no significant differences in seed-setting rate or 1000-grain weight across different nitrogen application rates, whereas nitrogen application significantly influenced effective panicle number and total grains per panicle. Therefore, the synergistic optimization of yield components through appropriate nitrogen fertilization is key to stabilizing rice production in low-temperature environments (Liu et al., 2019).

## 5 Conclusions

The results demonstrated that chilling treatment at the rice tillering and jointing stages affected the maturity date, grain yield, yield components and economic benefits, with more pronounced effects during the jointing stage. Nitrogen supplementation under low-temperature stress significantly improved rice tillering and panicle traits, simultaneously elevating grain yield and economic benefits. The single panicle weight, number of first branches and secondary branches, effective panicle number, total grain number per panicle, net income, and input-output ratio of rice all reached peak values at a nitrogen application rate of 267.90 kg·ha^-1^. Further increases in nitrogen application beyond this level resulted in a decline across all measured parameters. At this rate, yields under TLS and JLS conditions were 4424.40 kg·ha^-1^ and 2350.82 kg·ha^-1^, representing increases of 16.59%–102.04% and 11.09%–273.95%, respectively, compared to other nitrogen treatments. Among all nitrogen treatments, only the field control (CK) yielded positive net income under standard temperature conditions. The N_267.9_ treatment achieved a net income of 14,018.16 CNY·ha^-1^, exceeding the N_295.5_, N_240.3_, and N_212.7_ treatments by 29.60%, 31.11%, and 98.66%, respectively. In summary, a nitrogen application rate of 267.90 kg·ha^-1^ can effectively mitigate low-temperature damage in rice and serves as a recommended strategy for achieving synergistic improvements in both yield and economic return under chilling stress conditions. Future research should focus on elucidating the physiological and molecular mechanisms by which nitrogen regulates rice adaptation to low-temperature, providing a theoretical basis for climate-smart nitrogen management strategies.

## Data Availability

The original contributions presented in the study are included in the article/supplementary material. Further inquiries can be directed to the corresponding authors.
